# MicroRNAs and Growth Factors: An Alliance Propelling Tumor Progression

**DOI:** 10.3390/jcm4081578

**Published:** 2015-08-13

**Authors:** Merav Kedmi, Aldema Sas-Chen, Yosef Yarden

**Affiliations:** Department of Biological Regulation, Weizmann Institute of Science, Rehovot 76100, Israel; E-Mails: meravk@tlvmc.gov.il (M.K.); aldema.sas@weizmann.ac.il (A.S.-C.)

**Keywords:** cancer therapy, carcinoma, epidermal growth factor (EGF), metastasis, network, receptor tyrosine kinase, signal transduction, transcription

## Abstract

Tumor progression requires cancer cell proliferation, migration, invasion, and attraction of blood and lymph vessels. These processes are tightly regulated by growth factors and their intracellular signaling pathways, which culminate in transcriptional programs. Hence, oncogenic mutations often capture growth factor signaling, and drugs able to intercept the underlying biochemical routes might retard cancer spread. Along with messenger RNAs, microRNAs play regulatory roles in growth factor signaling and in tumor progression. Because growth factors regulate abundance of certain microRNAs and the latter modulate the abundance of proteins necessary for growth factor signaling, the two classes of molecules form a dense web of interactions, which are dominated by a few recurring modules. We review specific examples of the alliance formed by growth factors and microRNAs and refer primarily to the epidermal growth factor (EGF) pathway. Clinical applications of the crosstalk between microRNAs and growth factors are described, including relevance to cancer therapy and to emergence of resistance to specific drugs.

## 1. Introduction

Somatic mutations encompassing single base mutations, inter- and intrachromosomal rearrangements, as well as copy number changes are major initiators of the multistep process leading to malignancy. Germ line mutations, such as loss of tumor suppressor functions and the induction of oncogene functions facilitate somatic mutations [1,2], but the major driver of genetic aberrations is likely replication stress imposed by rapid divisions of stem cells and their immediate progenies [3,4]. The number of oncogenic (driver) mutations per common adult epithelial cancer is thought to exceed four aberrations [5], but fewer events are required in hematological cancers. On the way to become a metastatic tumor, the single initiated cancer cell must undergo rapid cell divisions, which fixate the oncogenic mutations, attract blood and lymph vessels that supply oxygen and nutrients, and invade the surrounding extracellular matrix and vessels, which permits dissemination and colonization in distant sites. This train of events is controlled by a plethora of tissue-specific growth factors [6]. For example, the 11 members of the epidermal growth factor (EGF) family act as both mitogens and motogens of epithelial cells, the precursors of carcinomas. The receptors for EGF family ligands and for other growth factors are typically transmembrane proteins sharing a tyrosine kinase catalytic function (called receptor tyrosine kinases, RTKs). Although growth factors are essential for progression of many solid tumors, accrual of specific oncogenic mutations might free cancer cells from their reliance on growth factors. This explains why a relatively large fraction of the genes undergoing recurrent somatic mutations in cancer affect protein kinases and other signaling proteins placed downstream of RTKs [7], such as B-RAF (in melanoma), RAS (in pancreatic cancer) ERBB2/HER2 (in breast cancer), and EGFR (in brain cancer). While the majority of tumors are characterized by enhanced secretion of growth factors (termed autocrine secretion [8]), driver mutations directly affecting growth factor genes are relatively rare. One example entails a platelet-derived growth factor gene fused to collagen, which is often found in dermatofibrosarcoma protuberans [9,10].

**Figure 1:**
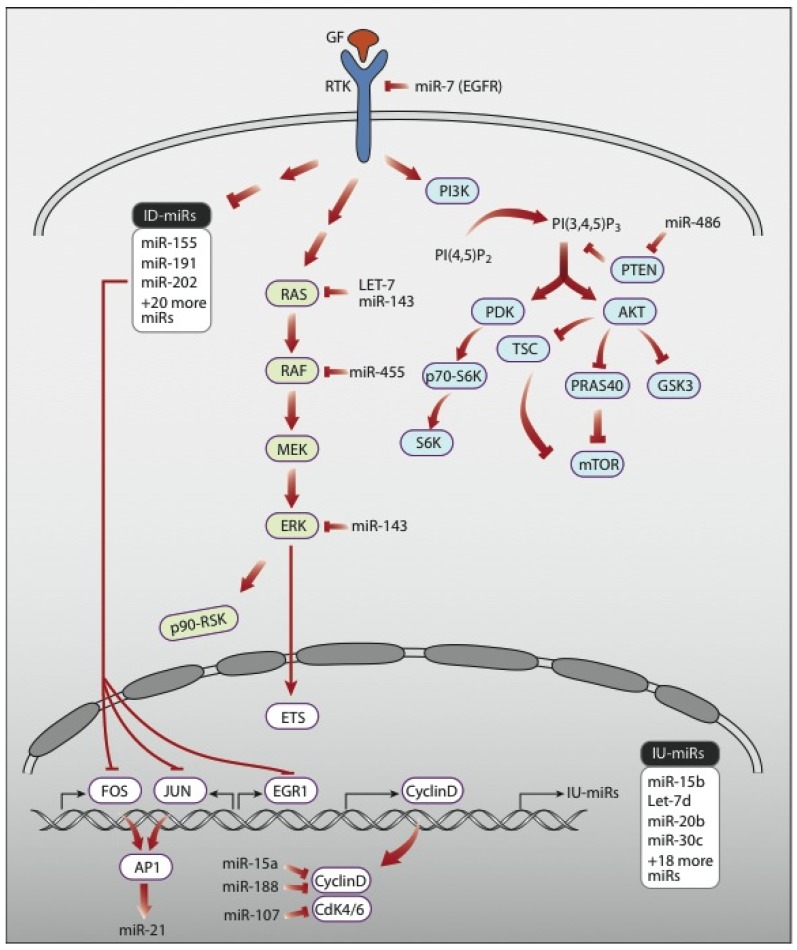
Schematic representation of RTK signaling pathways and representative regulatory microRNAs. Several biochemical signaling pathways are simultaneously activated upon binding of a growth factor (GF) to a receptor tyrosine kinase (RTK). Shown are two major cascades of protein kinases: the RAS-ERK pathway, which culminates in translocation of active ERK molecules to the nucleus, and the PI3K-to-AKT pathway, which requires phosphorylation of the inositol ring of phosphatitylinositol 4,5 bisphosphate at carbon position number 5. Both pathways regulate transcription factors, such as AP1, which comprises dimers of JUN and FOS. Note that many components of the signaling pathways are modulated at the mRNA level by microRNAs. Likewise, several microRNAs are induced or inhibited by RTK signals. They include a large group of microRNA molecules that undergo immediate down-regulation upon activation of EGFR (termed: ID-miRs) and several groups of microRNAs that are up-regulated immediately following RTK activation. For example, the group of IU-miRs is induced as early as 20 min after stimulation of EGFR.

Importantly, growth factors and their downstream signaling pathways propel not only tumor progression, but also survival of cancer cells under the intense stress imposed by chemotherapy and radiotherapy [6,11]. This broad spectrum of cellular outcomes is enabled by a cascade of biochemical events that transmit growth factor signals from an activated RTK, which undergoes rapid conformational alterations, followed by autophosphorylation [12] and recruitment of upstream adaptors, such as GRB2, SHC and IRS. Each adaptor instigates a vertical biochemical cascade. In the case of EGFR and its co-receptors, HER2, HER3, and HER4 (also called ERBB2 through ERBB4), the major cascades are the ERK mitogen-activated protein kinase (MAPK) pathway and the phosphatidylinositide 3-kinase (PI3K) route, leading to activation of the AKT kinase (see Figure 1). In addition to their cytoplasmic actions, the cascades initiated by RTKs lead to regulation of transcription of specific genes in the nucleus. This is often associated with movement of proteins into or out of the nucleus. For instance some MAPK substrates, including the E26 transformation specific (ETS) family member ERF, depart from the nucleus upon phosphorylation [13]. Similarly the FoxO family transcription factors, which are substrates for AKT, also leave the nucleus and therefore become inactive as transcription factors [14]. The first genes activated by a growth factor are typically seen to accumulate beginning approximately 20 min after the stimulus [15,16]. These early genes, called immediate early genes or IEGs, usually rise rapidly and then shortly after rising they quickly fall. Following the wave of IEGs, another set of genes, called the delayed early genes (or DEGs), some are negative regulators such as transcription repressors and MAPK phosphatases, are activated and like the IEGs they also rise and fall. Finally, approximately 2.5 h after stimulation, a third set of genes, termed late response genes, or LRGs, begins to rise. Unlike the IEGs and the DEGs, the LRGs do not drop in expression as long as the stimulus is maintained, but instead reach a steady state level of expression between 4 and 8 h after the stimulus [17].

**Figure 2:**
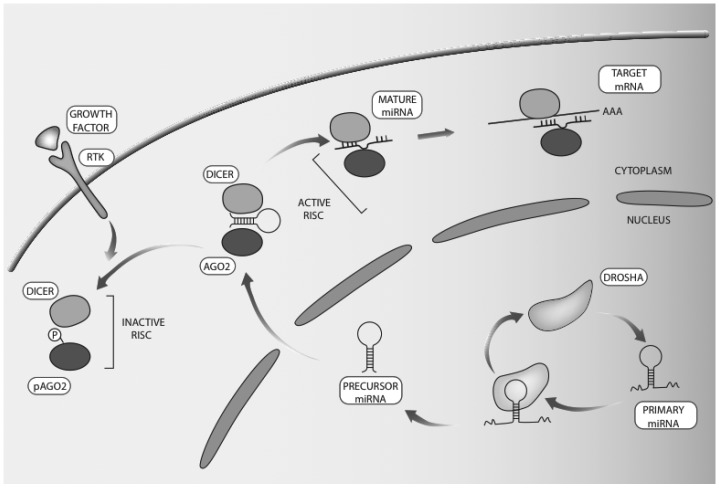
Schematic representation of microRNA biosynthesis and regulation by RTK signaling. microRNA biogenesis starts with transcription of the respective gene by RNA polymerase II. The formed long primary microRNA (pri-miRNA) consists of a hairpin stem, terminal loop and two single stranded regions. The RNase III endonuclease called Drosha processes pri-miRNAs into 70-nucleotide imperfect stem loop structures (pre-miRNAs). The latter are exported to the cytoplasm, to undergo processing by another RNase III endonuclease, Dicer, which removes the loop and joins the two arms. The resulting RNA duplex of 19–24 nucleotides allows one strand to be loaded into the RISC, while the other strand undergoes degradation. Mature microRNAs lead to translational repression or to mRNA degradation. Note that the RISC includes members of the Argonaute family, such as AGO2. It has been reported that phosphorylation of AGO2, at tyrosine 393, by EGFR is enhanced under hypoxia [32]. This is associated with dissociation of the AGO2-Drosha complex and with inhibition of processing of precursor microRNA molecules.

Similarly complex, wave-like patterns of expression might relate to microRNAs (miRNAs or miRs). miRNAs are distinguished by their size of 19–22 nucleotides, and a step-wise biogenesis pathway (see Figure 2). These relatively short RNA molecules are transcribed by RNA polymerase II as large primary transcripts (pri-miRs) that are processed by Drosha to yield 60–110 nucleotide long hairpins containing precursor miRNAs (pre-miRs) [18]. Following transport of the pre-miRs to the cytoplasm, mature miRNAs are excised from the pre-miRs by RNaseIII enzyme called Dicer [19] and loaded into the RNA-induced silencing complex (RISC) [20]. Once completed their maturation, miRNA molecules become competent to target mRNAs for decay or for translational arrest [21,22]. Targeting of an mRNA by a miRNA is mediated by base-pairing between nucleotides 2–8 of the miRNA and a target element in the transcript’s 3’ un-translated region (UTR) [23]. Because miRNAs are negative regulators of gene expression [24], and because each miRNA targets several hundreds of distinct mRNAs molecules [25], they greatly impact cellular processes involving de novo synthesis of proteins, such as tumor progression. This review highlights the cooperative interactions of miRNAs, their mRNA targets and growth factor signaling, in the context of tumor progression.

## 2. Occurrence and Biogenesis of microRNAs and Their Relevance to Cancer

According to the latest release of the miRBase database (release 21; June 2014), there are at least 2588 mature human microRNAs. miRNAs play profound roles in cancer progression, including metastasis. They can act both as oncogenes, namely, oncomiRs, and as tumor suppressor miRNAs. Changes in the abundance of specific miRNAs were demonstrated in many types of cancer, and their expression levels influence cell migration, invasion and proliferation [26]. Most of the miRNAs in cancer cells show down-regulated abundance compared to normal cells, however, several miRNAs are specifically up-regulated in cancer. In line with global alterations, it has been shown that malignant processes involve dysregulation or dysfunction of the miRNA biogenesis machinery due to mutations or epigenetic events (reviewed by [27] and by [28]). For example, expression of Drosha and/or Dicer is decreased in some tumor types, including neuroblastoma, liposarcoma, lung, breast, and ovarian cancers [29,30,31]. Growth factor signaling pathways, such as the epidermal growth factor receptor (EGFR) and the transforming growth factor beta (TGF-β) pathways, might affect general processing of miRNAs. EGFR restrains the maturation of specific tumor suppressor miRNAs, such as miR-31, -192, and miR-193a-5p, by phosphorylation of Argonaute 2 (AGO2) at Tyr393. This phosphorylation reduces the ability of Dicer to bind with AGO2, thereby inhibits processing of precursor miRNAs into mature miRNAs [32]. Under hypoxia, phosphorylation of Tyr393 by EGFR enhances cell survival and invasiveness and this was associated with poor prognosis of breast cancer patients [32]. TGF-β and bone morphogenic protein (BMP) signaling increases miR-21 abundance; specific SMAD signal transducers are recruited to the Drosha microprocessor complex and thus promote processing of primary miR-21 (pri-miR-21) into precursor miR-21 (pre-miR-21) [33]. Global effects of growth factors on miRNA biogenesis are associated in tumors with genomic rearrangements, which cause deletions or amplification of specific miRNAs loci. Conceivably, cancer cells make use of both growth factors and genetic aberrations to change miRNAs abundance, and consequently harness cellular machineries in favor of better adaptation to their changing environments.

## 3. Networks of Growth Factors and microRNAs

### 3.1. Growth Factors Regulating miRNAs

**Figure 3:**
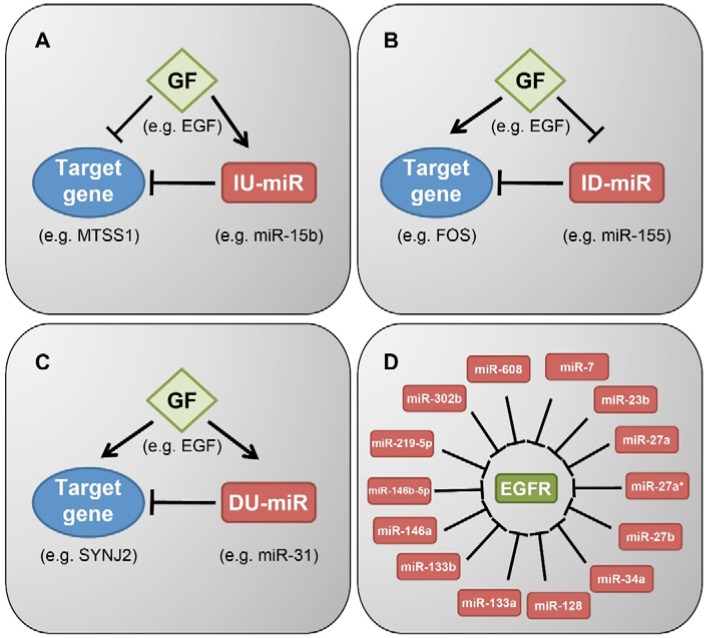
Network modules incorporating microRNAs and growth factor signaling pathways. (**A**) A feed-forward loop (FFL), whereby EGFR signaling down-regulates the expression of MTSS1, an inhibitor of metastasis. Inhibition of MTSS1 is strengthened by the induction of its targeting IU-miR, miR-15b; (**B**) A feed-forward loop whereby EGFR signaling up-regulates expression of the transcription factor FOS and, in parallel, down-regulates an ID-miR, miR-155, which inhibits *FOS*; (**C**) An incoherent FFL, whereby up-regulation of *SYNJ2*, a lipid phosphatase gene, by EGFR signaling is fine-tuned through the induction of the delayed up-regulated microRNA (DU-miR), miR-31, which inhibits *SYNJ2* expression; (**D**) Listed are miRNAs that directly target EGFR in different cancer cells. (ID-miR, immediately down-regulated miRNA; IU-miR, immediately up-regulated miRNA; DU-miR, delayed up-regulated miRNA).

Regulation of miRNA abundance might be induced, or otherwise influenced, by growth factors. Several studies analyzed changes in expression profiles of miRNAs following stimulation of cultured cells with specific growth factors. For instance, dynamic and coordinated changes in expression of groups of miRNAs were identified in normal mammary epithelial cells following stimulation with EGF. In less than 60 min post stimulation we observed both up- and down-regulation of distinct groups of miRNAs [34,35]. Interestingly the immediately down-regulated miRNAs we reported, a group consisting of 23 members, were over-represented among miRNAs that showed lower expression in breast cancer tumors compared to the surrounding normal tissue (peri-tumor) from the same patient [34]. Reciprocally, the up-regulated miRNAs were enriched among miRNAs with higher expression in the tumors [35]. Importantly, the mammary cells we tested, MCF10A, migrate in response to EGF stimulation [13,36]. Accordingly, we found that the migratory response of these cells is controlled by both up- and down-regulated miRNAs. For example, miR-15b, which was immediately up-regulated following EGF stimulation, significantly decelerated migration and invasion rates when silenced. In line with this observation, miR-15b expression was significantly higher in different breast cancer subtypes compared to control. MiR-15b’s novel target, metastasis suppressor 1 (*MTSS1*), a lipid-binder cytoskeletal protein, which is lost in some advanced tumors, was down-regulated following EGF treatment and mediated the effects on migration and invasion of normal and cancerous cells [35] (Figure 3A). Manipulation of the immediately down-regulated miRNAs following EGF stimulation also affected migration. Thus, silencing miR-191, which targets the immediate early gene called *EGR1*, elevated cell migration. Like miR-191, a significant number of the targets of immediately down-regulated miRNAs are IEGs, such as *FOS* and *JUN*. Under steady state, when EGF is not introduced to cells, the immediately down-regulated miRNAs (ID-miRs) inhibit the expression of the IEGs, some of which are proto-oncogenes. Correspondingly, upon EGF stimulation the expression levels of these miRNAs are decreased and a rapid up-regulation of the IEGs is achieved. For example, one of the ID-miRs, miR-155, directly targets *FOS* (Figure 3B). Interestingly, the oncogenic viral form of c*-FOS*, v-*FOS*, harbors a shorter 3′UTR than the c-*FOS* 3′UTR, which does not include miR-155’s target sequence. Hence, the transcript of v*-FOS* is not inhibited by miR-155, which allows v-*FOS* to exert its oncogenic ability [34]. In another comprehensive study, HeLa cells were stimulated with EGF for short times (15 min to 6 h) and miRNA expression levels were measured using microarray or deep-sequencing [37]. Dynamic changes in miRNA expression level were detected and the miRNA’s predicted targets were found to be involved in molecular functions that relate to EGF signaling, such as cellular development, proliferation, cell morphology, cell death, and cell-to-cell signaling and interaction [37].

Up regulation of three miRNAs, miR-31, miR-181b, and miR-222, was detected in oral cancer cells following treatment with EGF, and this was mediated by AKT and C/EBPβ signaling, at least in the case of miR-31 [38]. Increased expression of miR-31 was also observed in EGF-stimulated mammary cells [35]. MiR-31 directly targets synaptojanin 2 (SYNJ2), a lipid phosphatase transiently up-regulated following EGF treatment (Figure 3C). Hence, it is possible that miR-31 fine-tunes the expression of *SYNJ2*, meaning that it induces down-regulation of SYNJ2 back to baseline expression level. In patients with breast and brain cancer, *SYNJ2*’s high abundance was negatively correlated with miR-31 expression and associated with poor prognosis [39]. Congruently, forced expression of *SYNJ2* enhanced tumor growth and metastasis in mice, and increased formation of invadopodia and lamellipodia, actin-filled cellular extensions involved in invasion and migration, respectively [39].

The delayed response to EGF stimulation (3–12 h post stimulation) involves miRNAs targeting both apoptotic and anti-apoptotic genes. Specifically, miR-134, miR-145, miR-146b, miR-432, and miR-494 had the largest number of apoptotic and anti-apoptotic targets, including targets that are part of the interferon pathway [40]. Other miRNAs that were identified as regulators of apoptosis and are induced by growth factor receptors, such as EGFR and MET, are miR-221/222 and miR-30b/30c. It was further suggested that in response to treatment of lung cancer cells with tyrosine kinase inhibitors these miRNAs repress the pro-apoptotic genes *APAF1* and *BIM*, making cells less susceptible to apoptosis (reviewed by [41]). The miR-30 family has a tumor suppressive role. These family members were induced by SRC inhibitors and down-regulated by oncogenic growth factors signals such as EGF and the hepatocyte growth factor (HGF). Additionally, several of miR-30’s predicted targets, such as the MAPK-regulated transcription factor, ERG, are associated with epithelial-to-mesenchymal transition (EMT) [42]. In conclusion, several growth factor inducible miRNAs seem to act cooperatively to support survival, proliferation and motility, cellular functions vital for tumor progression.

### 3.2. Specific microRNAs Regulating Growth Factor Signaling

The other side of the miRNAs-growth factor networks is miRNAs that regulate the expression of growth factors, growth factor receptors, and their intracellular effectors. Specifically, we focus here on miRNAs that regulate EGFR, the EGFR pathway, and EGFR’s family members. EGFR itself can be regulated by multiple miRNAs. MiR-7 was one of the first miRNA identified as directly regulating EGFR. In glioblastoma, lung and breast cancer cells miR-7 blocked EGFR expression by means of accelerating mRNA decay. Potentially, miR-7 induces tumor suppressive actions by regulating not only EGFR but also the downstream signaling pathway at multiple sites. For example, this miRNA can inhibit AKT and ERK1/2 in several human cancer cell lines and it might decrease invasiveness and arrest the cell cycle [43,44]. Similarly, miR-128 was among the first miRNAs identified as an upstream regulator of EGFR. Interestingly, miR-128 loss of heterezygosity was frequently detected in lung cancer samples, in correlation with patient survival following treatment with an EGFR-specific TKI [45]. Other miRNAs that directly target EGFR include miR-23b/27b [46], miR-133a [47,48], miR-133b [49], miR-146a [50], miR-146b-5p [51], miR-219-5p [52], miR-302b [53] and miR-608 [54] (Figure 3D).

As initially exemplified by miR-7, other miRNAs can also target more than one component of the EGFR pathway. These include miR-124, miR-147, and miR-193a-3p, which inhibit G1/S transition and cell proliferation by targeting EGFR-driven cell cycle proteins [55]. MiR-143 and miR-145 target *KRAS*, *BRAF* [56] and *MEK2* [57] in colorectal cancer and also in other types of cancer such as prostate tumors [58]. MiR-27a (miR-27a-3p) and the complementary miR-27a* (miR-27a-5p), both targeting EGFR, were found to be significantly down-regulated in multiple head and neck squamous cell carcinoma cell lines. Interestingly, miR-27a* targets also AKT and mTOR (mammalian target of rapamycin) within the EGFR signaling pathway [59].

Other members of the EGFR/ERBB family are also regulated by miRNAs in cancer. Using miRNA gain-of-function screens and two HER2-amplified cell lines enabled identification of the following direct regulators of HER2: miR-552, miR-541, miR-193a-5p, miR-453, miR-134, miR-498, and miR-331-3p [60]. MiR-331-3p was found to target HER2 also in glioblastoma and prostate cancer cell lines [61,62]. In a similar way, miR-148b, miR-149, miR-326, and miR-520a-3p simultaneously down-regulated HER3/ERBB3 and components of the downstream signaling pathway in response to the direct ligand of HER3, neuregulin [63]. Interestingly, miR-125a and miR-125b target both HER2 and HER3 in breast cancer cells, and consequently inhibit phosphorylation of ERK and AKT [64]. miR-193a-3p directly targets HER4/ERBB4. Repression of HER4 by overexpression of miR-193a-3p resulted in decreased proliferation, migration, invasion and EMT, as well as increased apoptosis of lung cancer cells. Moreover, miR-193a-3p, which negatively regulates HER4 in xenograft tumor models, bears anti-tumor effects [65,66]. In esophageal squamous cell carcinoma, miR-302 targeted HER4, inhibited proliferation and invasion and induced apoptosis [67]. Taken together, it is conceivable that other subgroups of receptors for growth factors are regulated by multiple miRNAs, and the latter might coordinately control components of the downstream signaling pathway.

### 3.3. Feedback Loops Linking microRNAs and Growth Factors

The plot thickens. Bilateral regulation of growth factors and miRNAs in tumors generates high order complexity that is increasingly emerging now. Specifically, feedback regulatory loops in which a miRNA is both targeting a specific pathway and, at the same time, is regulated by the same pathway, likely confer module versatility and dynamicity. For example, several miRNAs that directly target EGFR are also regulated by EGFR signaling. Thus, miR-34a is up-regulated immediately following EGF stimulation [35], but it is also directly regulating EGFR. Possibly, through this complex regulation, miR-34a acts as a tumor suppressor in the development of chordoma [54]. As discussed above, miR-7 is a well-established regulator of EGFR, however it was also shown that miR-7 might be regulated by EGFR signaling: EGFR activation in lung cancer cells can stimulate miR-7 expression in an ERK-dependent manner, suggesting that EGFR induces miR-7 expression via the RAS-ERK pathway [68]. Feedback loops that involve specific miRNAs and different components of the EGFR pathway also exist: miR-143 and miR-145 regulate the EGFR pathway genes *KRAS*, *BRAF*, and *MEK2* [56,57], but EGFR signals down-regulated these tumor suppressor miRNAs in a murine model of colon cancer [69]. In addition, in lung cancer cells, EGFR down-regulated miR-145 expression through ERK1/2 [70]. Other, less direct feedback loops, were also identified, in which EGFR regulated the expression of miRNAs that in turn targeted other partners of the same pathway. For example, miR-21 expression levels are regulated by EGFR via the activation of beta-catenin and AP-1 [71], and miR-21 is suppressed by the EGFR inhibitor, AG1478, suggesting that the EGFR can regulate miR-21 expression [72]. On the other hand, miR-21 regulates EGFR and AKT signaling through VHL/beta-catenin and the PPARα/AP-1axis [71]. These networks of miRNAs and growth factors have roles to play in cells motility, proliferation, and other processes that involved in cancer pathogenesis and metastasis. It is therefore important to resolve these networks in a global and systemic way.

## 4. Potential Clinical Applications of miRNAs Relevant to Growth Factors and Signal Transduction

Because growth factor signaling is pivotal to tumor progression and it is often targeted by anti-cancer drugs, major efforts are being made with the aim of better classifying malignancies and improving diagnosis and personalized therapy [73]. Since the first identification of miRNA dysregulation in cancer [74], profiling the abundance of miRNAs in tumor samples [75,76], as well as in patient fluids [77,78], is increasingly implicated as a tool enabling improved diagnosis, prognosis, and assessment of therapeutic responses. For example, in the pioneering case of let-7, reduced expression of this miRNA in human lung tumors is associated with shortened postoperative survival [79]. The use of let-7 and other miRNAs as biomarkers has been facilitated by their high stability in patient samples [77,80]. Furthermore, expression of miRNAs has been shown to be highly tissue specific [81], such that miRNA profiling might be able to infer developmental origins of specific tumors [76,82].

In the last few years, measuring global patterns of miRNAs is becoming an established method for classifying individual tumors of breast [83,84,85,86], lung [87,88], and other organs [89]. Some of these classifications will likely reach clinical application. For example, a microRNA-based test that identifies the primary origin of 42 different types of tumors (denoted miRview-mets2) has been established [90]. The panel involves testing 64 miRNAs, previously validated in 489 specimens, including 146 metastatic tumors from 42 tissues of origin. The panel is based on a tree-classifier, originally developed by Rosenfeld and co-workers, who profiled 205 primary *versus* 131 metastatic tumors from 22 different tumor origins [82]. Another comprehensive study by Nair and co-workers reported meta-analysis of 43 miRNA profiling studies across 20 types of malignancies [91]. These authors argue that stringent standardization must be introduced into the process of miRNA profiling. In addition, they found that for all classifier miRNAs in studies that evaluated overall survival across diverse malignancies, the miRNAs most frequently associated with poor outcome were let-7 (decreased expression in patients with cancer) and miR-21 (increased expression). In the context of growth factor signaling, relative abundance of subsets of EGF-regulated miRNAs in breast cancer models have been shown to correlate with the abundance of miRNAs in breast cancer patients [34,35]. Thus, miRNA classifiers, especially those based on molecular mechanisms of disease, will likely evolve into major diagnostic and prognostic tools of cancer pathologists.

## 5. MicroRNAs as Molecular Targets of Future Cancer Therapeutics

Apart from their increasing role in classification and prognosis of cancer, miRNAs are emerging as potential targets of novel drugs. Since miRNAs can function both as oncogenes and tumor suppressors, two complementary therapeutic approaches relate to miRNA-mediated therapy: silencing the action of a specific miRNA or re-introducing a specific miRNA into patients (reviewed in [92,93]). Initial efforts concentrated on silencing expression of specific miRNAs. For example, Krutzfeldt and co-workers conducted intravenous administration of “antagomirs” to mice. Injection of antagomirs resulted in significant reduction in abundance of several miRNAs in various tissues (*i.e.*, miR-16, miR-122, miR-192, and miR-194) [94]. Since then, many delivery methods have been developed to systemically administer miRNA antisense oligonucleotides, which directly silence oncogenic miRNAs in tumors [95,96] or in the surrounding microenvironment [97]. Attenuation of miRNA action has also been achieved using miRNA sponges [98] and miR-masks [99], which contain sequences complementary to the miRNA target site or to the miRNA itself, respectively. On the other hand, replacement therapy focuses on re-expression of tumor suppressor miRNAs. Overcoming the loss of miRNA expression might be achieved either through introduction of synthetic miRNA mimics in the form of small double stranded and chemically modified oligonucleotides [100], or by using adenovirus-associated vectors (AAV), which do not stably integrate into the host genome [101].

Currently there are only three miRNA-targeting therapeutics in clinical trials, two of them in oncology. The first, miR-122, is an abundant liver-specific miRNA involved in the pathology of liver diseases, such as replication of hepatitis C virus (HCV) [102]. Since HCV infection depends on functional interactions between miR-122 and the HCV genome [103], miravirsen, a LNA-modified DNA phosphorothioate antisense oligonucleotide against miR-122, was developed as a potential drug [104]. Currently, miravirsen is applied in seven clinical trials, some are already in phase II. Another therapeutic endeavor employs MRX34, a liposome-formulated mimic of miR-34a, which is administered to patients with primary liver cancer or to those with liver metastasis from other cancers. Notably, miR-34a is embedded in the p53 transcriptional network [105]. Overexpression of miR-34a was found to inhibit tumor growth and to prolong survival of tumor-bearing mice [100,106,107]. A similar endeavor, which reached phase I trials, involves the tumor suppressor miR-16. Mice injected with miR-16 mimics showed dose-dependent inhibition of Malignant Pleural Mesothelioma (MPM) tumors [108]. This led to the development of TargomiRs, nanoparticles containing miR-16 mimics, which are administered to patients with MPM or with non-small cell lung cancer (NSCLC). Importantly, the nanoparticles are conjugated to anti-EGFR bi-specific antibodies that facilitate their targeted delivery to EGFR-expressing cells. Targeting microRNA-based therapeutics to cancer cells represents only one of many pharmacological challenges. These relate not only to drug efficacy, but also to potential toxicity due to the biology of miRNAs and their multiple targets.

## 6. MicroRNAs as Modulators of Patient Response to Drugs Targeting Growth Factor Signaling

Anti-cancer drugs able to intercept growth factor signaling currently outnumber other classes of therapies available to medical oncologists [109]. These drugs are effective on a broad range of carcinomas, and some drugs are active in more than one clinical indication, which is a rare situation in oncology. So far, only two classes of drugs have been approved: (i) monoclonal antibodies (mAbs), either naked or conjugated to a cytotoxic compound; and (ii) tyrosine kinase inhibitors (TKIs), which are either mono-specific or designed to inhibit several receptors. Along with weak efficacy, development of patient resistance to therapy hinders the effectiveness of both TKIs and mAbs. Mechanisms leading to resistance are only partially understood, and they include activation of surrogate pathways, acquired structural modifications of the drug target, and histological transformation, such as epithelial to mesenchymal transition (EMT) and small cell transformation [110]. Several studies point to potential roles of miRNAs in emergence of resistance to various cancer treatments, including chemotherapy [111], radiotherapy [112], and targeted therapy [113]. Below we discuss resistance to targeted therapy, as well as summarize experimental data relevant to clinical applications of mAbs against oncogenic receptors (Table 1) and small molecule TKIs (Table 2).

Overexpression of miR-7, a well-known regulator of EGFR expression [43], was shown to enhance the effect of an EGFR TKI, erlotinib, in a head and neck cancer model system [114]. Another receptor-targeting miRNA, miR-375, was shown to target the receptor for the insulin-like growth factor 1 (IGF1R) [115]. Importantly, miR-375 abundance negatively correlates to the expression of IGF1R in breast cancer specimens. Moreover, overexpression of miR-375 restored sensitivity to trastuzumab, an anti-HER2 mAb, and increased efficacy of trastuzumab in a xenograft model [115]. Sensitivity of breast cancer cells to trastuzumab was increased also by overexpression of miR-200c, a well-known miRNA regulating various cellular processes, including EMT [116]. Adam and co-workers reported that miR-200c is also associated with modulation of sensitivity of bladder carcinoma cell lines to an EGFR mAb, cetuximab [117]. Efficiency of cetuximab treatment was found to benefit also from concomitant overexpression of miR-146a: exposing hepatocellular carcinoma (HCC) cell lines to both cetuximab and miR-146a mimics elicited synergistic effects leading to increased apoptosis and decreased cell growth [118]. Like cetuximab, nimotuzumab binds to EGFR, and its inhibitory effects were enhanced by reducing abundance of miR-566 in glioblastoma cells [119]. The case of miR-221 is especially interesting as it was shown in two independent studies to facilitate the therapeutic effect of both trastuzumab [120] and gefitinib [121], in breast and lung cancer models, respectively. While miR-221 (along with miR-222) was shown to target APAF-1 in the lung model, its main target in the mammary model was the tumor suppressor PTEN. PTEN was also shown to be targeted by miR-21 [122]. This later report showed that overexpression of miR-21 decreases sensitivity of lung cells to gefitinib. Additionally, knock-down of miR-21 restored gefitinib sensitivity to a gefitinib-resistant lung cell line and caused a dramatic reduction in tumor size [122].

Interestingly, many studies relating to TKI-resistance focus on lung cancer models, and particularly on resistance to the EGFR-specific TKI called gefitinib. Accordingly, overexpression of miR-138-5p [123], miR-34a [124], miR-103 [121], or miR-203 [121] all resulted in increased sensitivity to gefitinib in lung cancer models, albeit by targeting different effectors. Conversely, in the case of miR-30b and miR-30c, inhibition of miRNA expression, rather than overexpression, increased drug sensitivity of gefitinib-resistant cells [121]. Likewise, overexpression of miR-203 was shown to enhance the effect of another EGFR-targeting TKI, CI-1033, and to reduce tumor size in a xenograft model of RAS-driven cells [125]. Collectively, the results we reviewed underscore potential roles for specific miRNAs in the acquisition of resistance to cancer drugs, and support the ability of yet other miRNAs to restore drug sensitivity. Therefore, targeting miRNAs in combination with conventional treatments may improve therapeutic efficacy of personalized cancer therapy.

**Table 1:** miRNAs modulating the efficacy of monoclonal antibodies targeting receptor tyrosine kinases.

miRNA	Target Gene(s)	Effect	Drug & Tumor	Reference
miR-566	VHL	Knockdown of miR-566 inhibited cell proliferation and invasion and led to cell cycle arrest in glioma cells. It further sensitized glioblastoma cells to Nimotuzumab	Nimotuzumab (glioblastoma)	[119]
miR-200c	ZNF217, ZEB1	Overexpression of miR-200c increased sensitivity to trastuzumab and suppressed invasiveness of breast cancer cell lines	Trastuzumab (breast)	[116]
miR-375	IGF1R	Overexpression of miR-375 restored sensitivity to trastuzumab resistant cell lines and increased the efficacy of trastuzumab in a xenogarft model.	Trastuzumab (breast)	[115]
miR-221	PTEN	Overexpression of miR-221 inhibited apoptosis, promoted metastasis and induced trastuzumab resistance in HER-2 positive breast cancer cells.	Trastuzumab (breast)	[120]
miR-200c	ERRFI-1	Overexpression of miR-200c regains sensitivity of the resistant cell lines to cetuximab treatment resulting in reduced cell growth *in vitro*	Cetuximab (bladder)	[117]
miR-146a	EGFR signaling	Overexpression of miR-146a suppressed cell growth and increased cellular apoptosis in HCC cell lines and displayed synergistic effects with cetuximab	Cetuximab (hepatocellular)	[118]
miR-7	EGFR	Overexpression of miR-7 enhanced the effect of erlotinib on growth inhibition of FaDu cells	Erlotinib (head&neck)	[114]

**Table 2:** miRNAs modulating efficacy of TKIs.

miRNA	Target Gene(s)	Effect	Drug & Tumor	Reference
miR-30b/30c and miR-221/222	BIM, APAF-1 (respectively)	Knockdown of miR-30b, -30c, -221 and -222 in gefitinib-resistant cells induces increased sensitivity gefitinib	Gefitinib (lung)	[121]
miR-21	PTEN	Overexpression of miR-21 decreased sensitivity of lung cells to gefitinib. Knock-down of miR-21 restored gefitinib sensitivity of the corresponding gefitinib-resistant cell line and caused a dramatic reduction in tumor size	Gefitinib (lung)	[122]
miR-34a	MET	Overexpression of miR-34a in EGFR mutant NSCLC increased sensitivity to gefitinib, resulting in increased inhibition of cell growth and to induced apoptosis, which resulted in tumor regression	Gefitinib (lung)	[124]
miR-138-5p	GPR124	Overexpression of miR-138-5p in NSCLC cells increased sensitivity to gefitinib *in vitro*	Gefitinib (lung)	[123]
miR-103 and miR-203	PKC-e, SRC (respectively)	Overexpression of miR-103 and miR-203 increased sensitivity to gefitinib in lung cells resistant to the drug	Gefitinib (lung)	[121]
miR-203	EREG, TGFA, API5, BIRC2, TRIAP1	Overexpression of miR-203 synergistically enhanced the effect of CI-1033 on reduction of tumor size in a xenograft model of nude mice injected with Ras-activated cells	CI-1033 (prostate)	[125]

## 7. Concluding Remarks

MicroRNAs can target growth factor pathways and, vice versa, growth factor pathways can regulate miRNA biogenesis. This bilateral crosstalk creates complex networks that are involved in multiple sub-programs of tumor progression, such as cell cycle regulation, EMT, and metastasis. Moreover, as we discussed herein, the complexity and robustness of these networks are enhanced by recurring feedback regulatory modules. As expected, miRNAs and growth factor signals are embedded in larger regulatory networks that control patient response to therapeutic interventions, such as monoclonal antibodies. It is therefore essential to resolve miRNA networks at high granularity and understand their functional logic. Thus, comprehensive mapping and understanding of the miRNA and growth factor alliance holds promise in terms of more effective cancer treatments that avoid emergence of patient resistance. Other future applications might include utilization of the miRNAs-growth factor networks as classifiers of cancer subtypes and markers for cancer diagnostics and prognosis.
